# Different theta connectivity patterns underlie pleasantness evoked by familiar and unfamiliar music

**DOI:** 10.1038/s41598-021-98033-5

**Published:** 2021-09-17

**Authors:** Alberto Ara, Josep Marco-Pallarés

**Affiliations:** 1 Department of Cognition, Development and Educational Psychology, Institute of Neurosciences, University of Barcelona, Barcelona, Spain; 2 Cognition and Brain Plasticity Unit, Bellvitge Biomedical Research Institute (IDIBELL), L’Hospitalet de Llobregat, Barcelona, Spain

**Keywords:** Auditory system, Cognitive neuroscience, Emotion, Reward

## Abstract

Music-evoked pleasantness has been extensively reported to be modulated by familiarity. Nevertheless, while the brain temporal dynamics underlying the process of giving value to music are beginning to be understood, little is known about how familiarity might modulate the oscillatory activity associated with music-evoked pleasantness. The goal of the present experiment was to study the influence of familiarity in the relation between theta phase synchronization and music-evoked pleasantness. EEG was recorded from 22 healthy participants while they were listening to both familiar and unfamiliar music and rating the experienced degree of evoked pleasantness. By exploring interactions, we found that right fronto-temporal theta synchronization was positively associated with music-evoked pleasantness when listening to unfamiliar music. On the contrary, inter-hemispheric temporo-parietal theta synchronization was positively associated with music-evoked pleasantness when listening to familiar music. These results shed some light on the possible oscillatory mechanisms underlying fronto-temporal and temporo-parietal connectivity and their relationship with music-evoked pleasantness and familiarity.

## Introduction

Music is undoubtedly a powerful source of pleasure for most human beings, despite its abstract nature. While multiple factors may contribute to music’s hedonic impact, its relation to familiarity has drawn the attention of researchers across disciplines. Evidence suggests a trend in preferring familiar music over unfamiliar one^1^, indicating that familiarity is an important modulator of music-evoked pleasantness. Classic studies in experimental psychology had described a “mere exposure” effect on musical appreciation, consisting in preference for music that had been previously listened to due to implicit psychological mechanisms^2^. This opened up a line of research across disciplines that has since delved into what is known about the concrete cognitive and affective phenomena behind this effect^1^. Music-evoked pleasantness as a function of familiarity has been classically attributed to a reduction in perceived complexity. In other words, it is hypothesized that with repeated exposure, the listener implicitly learns the attributes and contingencies of the stimuli and therefore perceives them as less complex and more agreeable^3^. Indeed, some theories propose that when individuals listen to music, they make predictions about how it is going to unfold based on both schematic expectations (i.e. implicit knowledge about the encultured rules of music) and veridical expectations (i.e. factual knowledge about concrete pieces of music)^4–6^. When these predictions are compared to the actual incoming information, reward signals are believed to be triggered as a function of the certainty of the predictions and the surprisal of the outcomes^5^. In the case of music-evoked pleasantness as a function of factual familiarity, the most pleasurable musical events would be characterized by a compromise between predictability and surprise^6^. As individuals are exposed to music, predictions are assumed to be updated, and so is their associated hedonic value. According to this view, familiarity effects on music-evoked pleasantness could be explained by an increase in predictive precision as veridical expectations come into play, at least while there is still room for surprise. In other words, more accurate knowledge about how music will unfold is hypothesized to generate better predictions that lead to more pleasurable surprisal/realization dynamics.

Consistent with this theoretical account, neuroscientific research has associated the pleasurable experience of listening to music with predictive coding over the auditory domain via fronto-temporal loops^5^, as well as with the brain reward system via dopaminergic activation of the striatum^7^. The different nodes comprising this network carry out relevant functions for music perception to take place. On the one hand, the temporal lobe is crucial in the processing of auditory inputs^8^. On the other hand, the prefrontal cortex is a pivotal area for emotional control^9^, the processing of time information^10^ and working memory^11^ in the musical domain. Functional streams assembling these two structures have been hypothesized to underly auditory working memory, which in turn enables the predictive dynamics ultimately necessary for music to become rewarding^12^. Indeed, temporal areas such as the superior temporal gyrus and Heschl’s gyrus along prefrontal areas such as the ventromedial prefrontal cortex, orbitofrontal cortex and the inferior frontal gyrus all have been related to pleasant music listening^13–16^. Moreover, both structural and functional connectivity between temporal and frontal nodes and the striatum have been found to be modulated by music-evoked pleasantness and individual differences in music-reward sensitivity^17–19^.

The oscillatory dynamics of the brain interactions underlying music-evoked pleasantness have also been studied. Most notoriously, both frontal theta power and fronto-temporal theta synchronization have been related to music-evoked pleasantness and related constructs^9,20–24^. Interestingly, frontal and fronto-temporal theta rhythms have also been related to working memory and expectancy in the auditory and musical domains^25,26^, suggesting further evidence in favor of the predictive coding hypothesis of music-evoked pleasantness.

In addition to this corpus of evidence, the neural underpinnings underlying preference for (factually) familiar music are also beginning to be understood. Among the few, an fMRI study by Green et al.^27^ linked listening to previously exposed music during a pleasantness task to an increase of activity in the dorsolateral prefrontal cortex and the inferior parietal cortex. The authors interpreted this functional interplay as underlying unintentional memory retrieval of familiar content into working memory upon re-exposure. Regarding music-related familiarity, Jagiello et al.^28^ also found greater late amplitudes over frontal and parietal topographies to be related to familiar music listening in an ERP study, phenomena related to recognition processes elsewhere^29^. Moreover, in a metanalysis by Freitas et al.^30^, listening to familiar music was found to be associated with activity in the left superior frontal gyrus, the ventral lateral nucleus of the left thalamus and the left medial frontal gyrus; while listening to unfamiliar music was associated to activity in the left insula, right cingulate cortex and right middle frontal gyrus. Interestingly, there is a great amount of overlap between the engaged areas found in these studies and the areas consistently found to be related to music-evoked pleasantness in other lines of research, particularly over frontal cortices. This would support the idea that familiarity and evoked pleasantness are indeed interlaced during music listening. Nonetheless, some degree of topological differentiation is also observed, suggesting that other brain mechanisms may come into play when listening to pleasant familiar music.

While the literature commented above identifies the spatial and temporal signatures of the studied phenomenon, these fMRI and ERP studies do not tackle the neural mechanisms that might explain how the different brain areas underlying pleasantness evoked by familiar music bind together. In these regards, neural oscillations have been hypothesized to be the means of communication between the different nodes of a brain network underlying cognition, with slow rhythms particularly well suited to synchronize distant brain areas^31^. To date, however, the connectivity dynamics between the brain nodes associated with pleasantness evoked by familiar music have not been addressed, neither their oscillatory signature.

In the present experiment we studied the slow cortical rhythms associated with music-evoked pleasantness as modulated by familiarity. In order to reach a good compromise between ecologic validity and experimental control, we induced familiarity in an exposure session using (likely) unknown music stimuli that otherwise were naturalistic and conforming to the participants’ preferences. Motivated by a connectivity approach and based on the results of previous studies^24,27,28^ and physiological plausibility^31^, we focused our analysis on theta synchronization between frontal, temporal and parietal signals. We hypothesized that the association between fronto-temporal theta synchronization and music-evoked pleasantness would be modulated by familiarity. In addition, we expected other connectivity topologies to show up as a result of taking familiarity into account.

## Materials and methods

### Participants

Twenty-two right-handed individuals (M = 21.86 years old, SD = 2.36, 17 women) participated in the experiment. The sample consists in a subset of the participants in Ref.^24^, in which we described the EEG data of the exposure session (see “Experimental procedure” section), not reported in the current manuscript. Recruitment was made through advertisement at the university campus. All participants were chosen to roughly have similar music preferences toward indie, pop, electronic and folk music genres as assessed with the Short Test of Music Preferences revised (STOMP-R, cut-off ≥ 4)^32^ as well as similar profiles of music reward and physical anhedonia as assessed with the Barcelona Music Reward Questionnaire (BRMQ, cut-off > 64)^33^ and the Physical Anhedonia Scale (PAS, males cut-off < 28, females cut-off < 20)^34^, respectively. None of the participant had received formal training in music for more than 3 years. All participants gave written informed consent and were paid 10€ per hour. All procedures were approved by the Bioethical Commission of the University of Barcelona and all experimental procedures were carried out according to the relevant guidelines and regulations.

### Stimuli

The musical stimuli used in this study were the same as in Ara and Marco-Pallarés^24^. Sixty musical fragments formed a pool of stimuli from which the experimental excerpts were taken. The stimuli consisted in fragments of 45 s from commercially available songs of several music genres including indie, pop, electronic, folk and experimental music (see Table S1 in the Supplementary Materials for the complete list of songs and the respective passages contained in the excerpts). These stimuli were selected to be likely unfamiliar and to elicit variable degrees of pleasantness based on the results of a pilot study with a separate sample of individuals. The 45 s fragments were chosen to be representative of the whole musical pieces (e.g. that they included more than one theme, that variations took place and/or that several instruments were present).

### Experimental procedure

**Figure 1:**
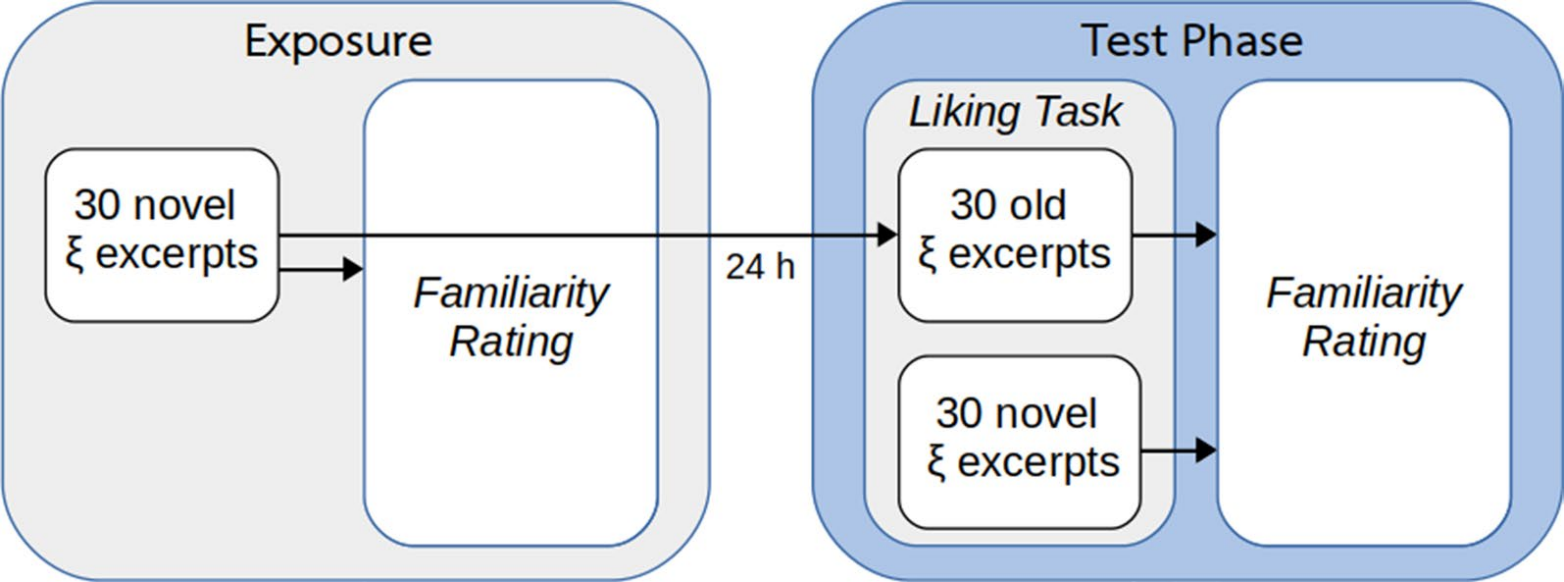
Experimental design diagram. During exposure, participants listened to 30 music stimuli randomly taken from a pool of 60 stimuli and rated their familiarity with the materials after each excerpt had been presented. Twenty-four hours later, participants listened to the same stimuli plus another 30 novel ones while continuously reporting the degree of pleasantness evoked. After each excerpt had been presented, participants rated their familiarity with the materials.

The experimental design is depicted as a diagram in Fig. 1. The experiment was divided in two sessions with a 24 h inter-session lapse: an exposure session and an experimental session. In the exposure session, participants were exposed to 30 music excerpts randomly drawn from the pool of stimuli. After each excerpt had finished participants responded to a 7-point Likert familiarity scale, where 1 meant “I had never heard this song before”, 2 meant “it sounds familiar but I cannot recognize it”, 3 meant “I have listened to this song once”, 4 meant “I have listened to this song a few times”, 5 meant “I have listened to this son numerous times”, 6 meant “I have listened to this song a lot” and 7 meant “I know this song to perfection”. In the experimental session participants listened to the same materials plus a set of 30 novel excerpts, in random order, and were asked to rate the degree of evoked pleasantness on a continuous basis while listening to each excerpt with as many responses as they wanted, following the same procedure used in Ref.^24^. Responses were given via the numeric keys of a computer keyboard with the following equivalences: 1: “I don’t like it”; 2: “I like it a little”; 3: “I like it moderately”; 4: “I like it a lot”; and 5: “I experience frissons”. Response keys had to be held for as long as a particular rating applied for the individual. Participants had to look to a fixation cross while listening to the excerpts. If no response was given after half the stimulus was presented, that trial was halted and automatically rejected from all conditions for that subject. After each excerpt had finished participants responded to the same Likert familiarity scale.

### Self-reported data

In order to have a metric index of online evoked pleasantness for each trial in the experimental session we computed the average of every response given for each excerpt weighted by the amount of time each response was held, following the same formulation used in Ref.^24^. For the statistical analysis of the EEG data, these data were dichotomized per subject by the means of a median split in order to ease model-fit and interpretation (excerpts rated below the median were coded as “least pleasant”, while excerpts rated equally or above the median were coded as “most pleasant”). In order to make sure that we included in subsequent analysis only excerpts that were unfamiliar the first time participants listened to them, we rejected those trials where music was rated with a value greater than 2 in the familiarity scale in the exposure session and in the novel condition of the experimental session from all conditions of the respective participant’s data set (rejection cut-off > 2).

### EEG data acquisition

Similarly to Ref.^24^, EEG was recorded from the scalp during the second session (0.01 Hz high-pass filter with a notch filter at 50 Hz; 250 Hz sampling rate) using a BrainAmp amplifier with tin electrodes mounted on an Easycap (Brain Products), at 61 standard positions (Fp1/2, AF3/4, Fz, F7/8, F5/6 F3/4, F1/2, FCz, FT9/10, FT7/8, FC5/6, FC3/4, FC1/2, Cz, T7/8, C5/C6, C3/4, C1/2, CPz, TP9/10, TP7/8, CP5/6, CP3/4, CP1/2, Pz, P7/8, P5/6, P3/4, P2/1, POz, PO7/8, PO3/4, Oz, O1/2) and left and right mastoids. An electrode placed at the lateral outer canthus of the right eye served as an on-line reference and an electrode at the infraorbital ridge of the right eye was used to monitor vertical eye movements. Electrode impedances were kept below 10 kΩ during the whole session.

### EEG signal processing

The same signal processing procedures used in Ara and Marco-Pallarés^24^ were applied to this study’s EEG data. EEG was re-referenced off-line to the linked mastoids and band-pass filtered from 0.1 to 45 Hz. Epochs consisted in the whole time-window of each listening and were baseline-corrected using the average of the whole fragment. Artifacts in these epochs were identified and corrected using independent component analysis (ICA). Epochs with absolute mean amplitude higher than 100 μV after ICA correction were rejected. Together with trials rejected for containing familiar music for the participants, the average proportion of rejected trials was M = 15.64% ± SD = 8.49%. One subject was excluded from the analysis because of poor physiological data quality. The surface Laplacian transform was applied to these data in order to reduce volume conduction and make the data reference-free^35^. To avoid attentional effects on the EEG at the beginning and end of the songs, the first and last 2 s were removed from the epochs for subsequent analysis. Time–frequency decomposition was computed on each epoch using 5-cycle complex Morlet wavelets in the frequency band of interest (θ: 4–8 Hz). Phase values for each electrode and frequency were obtained over time from this decomposition.

Then, ISPC-time was computed for each epoch as an index of phase synchronization between signals. This index describes the consistency in phase difference between two signals over time and is defined as:

1 $${\mathrm{ISPC}}_{\mathrm{f}}=\left|{\mathrm{n}}^{-1}{\sum }_{\mathrm{t}=1}^{\mathrm{n}}{\mathrm{e}}^{\mathrm{i}\left({\upphi }_{\mathrm{it}}-{\upphi }_{\mathrm{jt}}\right)}\right|,$$

where f is a given frequency, n is the number of time points and Φ_it_ and Φ_jt_ are the phases of two given electrodes at a given time point^36^. This was done for every frequency in the band of interest and all electrode pairs involving frontal, temporal and parietal signals (AF3/4, Fz, F7/8, F5/6 F3/4, F1/2, FCz, FT7/8, FC5/6, FC3/4, FC1/2, Cz, T7/8, TP7/8, CP5/6, CP3/4, CP1/2, Pz, P7/8, P5/6, P3/4, P2/1, POz, PO7/8, PO3/4). Finally, ISPCs were averaged across frequencies. We excluded from subsequent analysis connections involving peripheral electrodes (Fp1/2, FT9/10, TP9/10) and connections where the two electrodes were less than 6 cm apart from each other, since these most likely reflect residual artifactual activity and volume conduction, respectively.

### Statistical analysis

#### Self-reported data

In order to investigate whether participants found target music (i.e. old music in the experimental session) presented in the experimental session more familiar than in the exposure session and as compared to novel music in the experimental session a Bayesian multilevel ordinal regression model was carried out with reported familiarity as response variable, condition as explanatory variable and varying intercepts and slopes per subject. A cumulative likelihood function with the probit link function was assumed to explain the data in order to treat the Likert scale as ordinal (μ = probit(x), σ = 1). Weakly informative priors were placed over the latent variable’s thresholds and slopes (normal: μ = 0, σ = 1), as well as over the varying effects (gamma: α = 2, β = 2). The reference explanatory condition was target music in the experimental session (old music). Therefore, the difference between target music in the experimental session and in the exposure session was quantified by coefficient β_1_. The difference between old music and novel music in the experimental session was quantified by coefficient β_2_. The difference between novel music in the experimental session and target music in the exposure session was quantified by the difference between slopes (β_2_ − β_1_). To test the group-level differences to be non-zero a 95% highest density interval (HDI) was used to check the inclusion of the null hypotheses (H_0_: β_1_, β_2_, β_2_ − β_1_ = 0) in the posteriors assuming a region of practical equivalence (ROPE) of ± 0.01^37^. The reported point estimates correspond to the mode of the posteriors.

In order to investigate the difference between conditions of the experimental session (new vs. old) in reported pleasantness, a generalized Bayesian multilevel linear model was carried out with reported pleasantness as response variable, condition as explanatory variable and varying intercepts and slopes per subject. A student-t likelihood function was assumed to explain the data in order to accommodate outliers (μ = identity; prior on σ: student-t: μ = 0, σ = 10, ν = 3; prior on ν: gamma: α = 2, β = 0.1). Weakly informative priors were placed over the intercept and slope (normal: μ = 0, σ = 1), as well as over the varying effects (gamma: α = 2, β = 2). To test the group-level slope to be non-zero a 95% HDI was used to check the inclusion of the null hypothesis (H_0_: β_1_ = 0) in the posterior assuming a ROPE of ± 0.01. The reported point estimate corresponds to the mode of the posterior and quantifies the difference between conditions with new music as the reference explanatory condition.

### EEG phase synchronization

In order to investigate the effects of music-evoked pleasantness on theta synchronization as moderated by familiarity in the experimental session, mass-univariate Bayesian multilevel beta regression models were carried out with ISPCs as response variables, dichotomous pleasantness, familiarity condition and their interaction as explanatory variables and varying intercepts and slopes per subject. A beta likelihood function with the logit link function was assumed to explain the data since ISPC-time values are non-normally distributed in the unit interval (μ = logit(x); prior on ϕ: gamma: α = 0.01, β = 0.01). Weakly informative priors were placed over the overall intercepts and slopes (normal: μ = 0, σ = 1), as well as over the varying effects (gamma: α = 2, β = 2). The moderation effects of familiarity on the relationship between pleasantness and ISPCs were quantified by coefficients β_3_. To test the group-level moderation effects to be non-zero a 95% HDI was used to check the inclusion of the null hypotheses (H_0_: β_3_ = 0) in the posteriors assuming a ROPE of ± 0.01. When a non-zero interaction was found, post-hoc posterior inspection was carried out according to the model in order to explore non-zero differences between least pleasant and most pleasant reports in each familiarity condition (H_0_: PH = 0; PH_new_most-new_least_ = β_1_, PH_old_most-old_least_ = β_1_ + β_3_) with a 95% HDI assuming a ROPE of ± 0.01. Only connections exhibiting non-zero results in these post-hoc explorations are considered. Reported point estimates correspond to the mode of the posteriors.

### Bayesian inference specification

We used Bayesian inference in order to ameliorate the multiple testing problem posed by mass-univariate analyses without having to employ arbitrary thresholds nor the overly restrictive post-hoc corrections usually associated with frequentist statistics. This is achieved by virtue of Bayesian inference’s statistical properties (see Ara and Marco-Pallarés^24^ for a similar case). In addition, the generalized linear model allowed us to make appropriate assumptions about the dependent variables’ distributions.

Posterior distributions were approximated using 5 markov chains of 2000 samples with no thinning, burning-in the first 1000 samples. The No-U-turn sampler algorithm was used to draw samples. All chains were initialized at 0. All models converged as indicated by Gelman’s split-R-hat equaling 1^38^.

## Results

### Self-reported data

**Figure 2:**
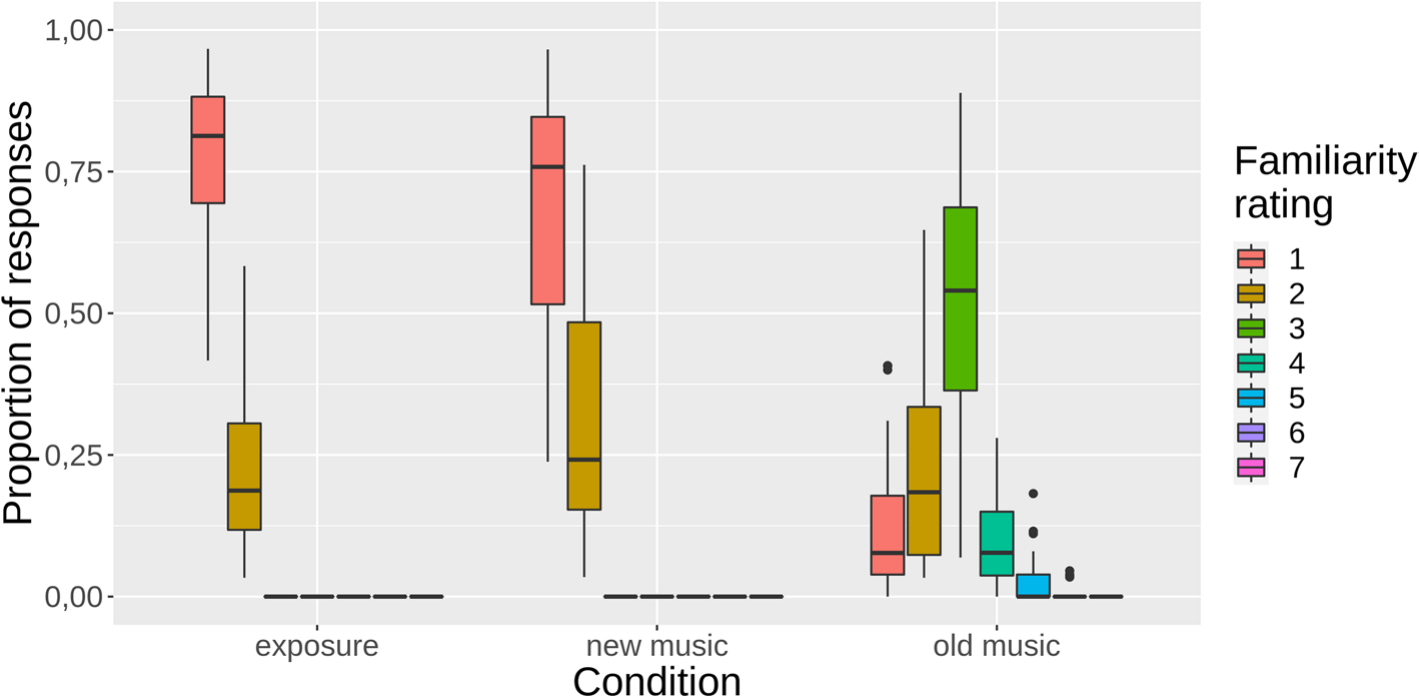
Distribution of average proportion of responses given for each familiarity rating across subjects in each familiarity condition (1: “I had never heard this song before”; 2: “it sounds familiar but I cannot recognize it”; 3: “I have listened to this song once”; 4: “I have listened to this song a few times”; 5: “I have listened to this son numerous times”, 6: “I have listened to this song a lot” and 7: “I know this song to perfection”). Music recognized as familiar in the exposure session and in the new music condition of the experimental session are excluded from all conditions (rejection cutoff > 2). Plot generated in R^53^ with package ggplot2^54^.

Figure 2 shows the familiarity ratings for the excerpts listened the first day (exposure), their repetition the second day (old music) and the new songs listened the second day (new music). As can be seen, ratings for the exposure and new music were mainly 1 (“I had never heard this song before”) or 2 (“it sounds familiar but I cannot recognize it”), while for the old music the most selected rating was 3 (“I have listened to this song once”). This was further corroborated by the statistical analysis showing that familiarity ratings were higher for target music in the experimental session than in the exposure session (β_1_ =  − 2.34, 95% HDI =  − 2.67 to [− 2.02]) and as compared to novel music’s in the experimental session (β_2_ =  − 2.14, 95% HDI =  − 2.46 to [− 1.79]; coefficients are expressed in standard deviation units). These results show that, overall, participants significantly recognized the songs played the day before. The distribution of familiarity ratings per excerpt the first time they were listened to (exposure session) is displayed in Fig. S1 of the Supplementary Materials.

**Figure 3:**
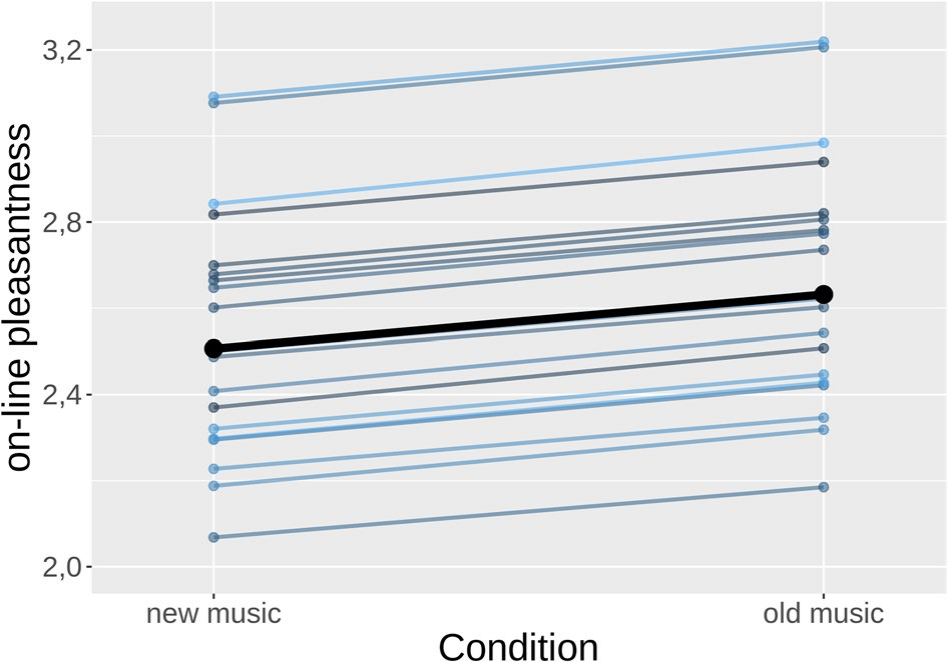
Time-weighted self-reported pleasantness in the two familiarity conditions of the experimental session (new vs old). Thin blue lines represent each participant’s increase. The thick black line represents the group-level increase. Dots represent parameter estimates. Plot generated in R^53^ with package ggplot2^54^.

In addition, time-weighted reported pleasantness was higher for old songs than for new songs in the experimental session (β_1_ = 0.12, 95% HDI = 0.01–0.24). Figure 3 shows that pleasantness reported by all the participants was higher for the excerpts that were previously listened to than for the new ones. The distribution of time-weighted reported pleasantness per excerpt and subject is displayed in Fig. S2 of the Supplementary Materials.

### EEG phase synchronization

**Figure 4:**
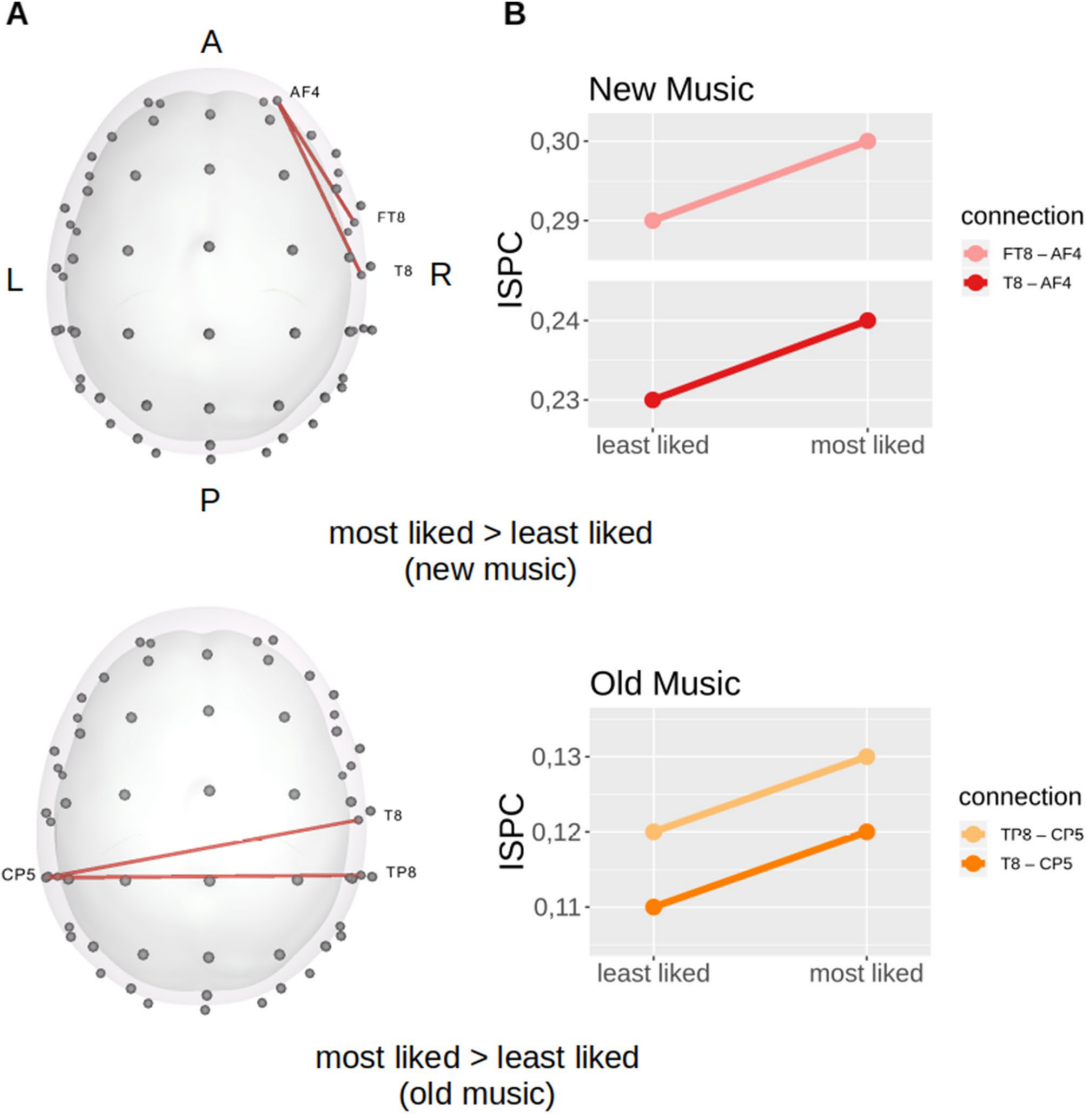
Non-zero results **(A)** with their corresponding prediction plots **(B)** in the theta connections of interest. Straight lines represent predictions of the response variables in each pleasantness $\otimes$  familiarity condition of interest following parameter estimates. Predictions are made on the original scale of the dependent variable. Prediction plots generated in R^53^ with package ggplot2^54^.

Two right fronto-temporal connections exhibited a non-zero increase with reported pleasantness in the new condition only (AF4-FT8: β_3_ =  − 0.08, 95% HDI =  − 0.13 to [− 0.02], PH_new_most-new_least_ = 0.05, 95% HDI = 0.02—0.09; AF4-T8: β_3_ =  − 0.08, 95% HDI =  − 0.13 to [− 0.02], PH_new_most-new_least_ = 0.06, 95% HDI = 0.01–0.10). In addition, two right-temporal to left-parietal connections exhibited a non-zero increase with reported pleasantness in the old condition only (T8-CP5: β_3_ = 0.14, 95% HDI = 0.06–0.22, PH_old_most-old_least_ = 0.08, 95% HDI = 0.01–0.14; CP5-TP8: β_3_ = 0.16, 95% HDI = 0.07–0.24, PH_old_most-old_least_ = 0.10, 95% HDI = 0.03–0.16). Coefficients are expressed in log-odds. Results are displayed in Fig. 4. In order to compare these results with standard frequentist approaches using different alpha levels please see Fig. S3 in the Supplementary Materials.

## Discussion

The goal of the present experiment was to study effects of familiarity and music-evoked pleasantness on phase synchronization between frontal, temporal and parietal signals in the theta oscillatory band. We exposed a cohort of participants to a set of musical fragments and measured their EEG signals and evoked reported pleasantness 24 h later in a second listening session, along a set of novel musical fragments.

Familiarity ratings for target stimuli were significantly greater in the experimental session than in the exposure session and as compared to novel stimuli in the experimental session, indicating that familiarity was indeed induced. Consistent with previous literature, pleasantness evoked by old songs was significantly greater than for new songs, revealing that one repetition and 24 h of consolidation was enough to induce familiarity effects of on music-evoked pleasantness. Seminal studies had already demonstrated that this effect is observed with as few as one repetition, even when explicit recognition fails^2,39^. In the context of the current study, we consider the presence of familiarity effects relevant, since it demonstrates that familiarity was not only induced, but also interlaced with self-reported pleasantness, thus making subsequent analysis of EEG data pertinent.

In the EEG synchronization analysis, we observed an increase in right fronto-temporal theta synchronization with greater reported pleasantness for unfamiliar musical fragments. Conversely, pleasantness evoked by familiar music revealed greater theta synchronization between right temporal and left parietal signals. These results suggest that different theta connectivity patterns are involved in the process of giving value to music depending on whether the stimuli are familiar or unfamiliar to the listener.

Right fronto-temporal cortices have been found to co-activate during pleasant music listening and to be related to reward-processing areas during peak pleasurable events^13,17^. Furthermore, frontal and parietal areas have been related to emotional control in reaction to music^9^. Fronto-temporal loops also underly working memory and PE processing in the auditory and musical domains^25,26^, functions hypothesized to be involved in music-evoked pleasantness by enabling the temporal representations and predictive dynamics necessary for music perception and its subsequent affective evaluation^5^.

In addition, theta oscillations have been revealed to play a role in music-evoked pleasantness. Frontal theta power has been associated with music-evoked pleasantness and related constructs such as evoked positive valence and musical consonance^9,20–23^. Importantly, right fronto-temporal theta synchronization in similar areas to the ones found in Ara and Marco-Pallarés^24^ was observed to increase with reported pleasantness when listening to novel music. These consistent results suggest that music-evoked pleasantness depends on right fronto-temporal connectivity when the musical stimuli are unknown, possibly due to the processing of schematic expectations while music is unfolding within auditory working memory.

On the other hand, frontal and parietal cortices have been reported to correlate with music-induced familiarity. This functional topology has been interpreted to underlie memory retrieval of known musical materials elsewhere^27^. Likewise, EEG activity over right frontal and left parietal regions has been related to familiar music listening^28^ and left temporo-parietal theta activity is thought to be involved in recognition memory^40^. We found theta synchronization between right temporal and left parietal signals to increase with pleasantness evoked by familiar music. This association suggests that music-evoked pleasantness relies more strongly on the neuropsychological mechanisms emerging from these connections when the musical stimuli are known, possibly recognition processing in the form of veridical expectations over the auditory domain.

It is difficult to determine from these results whether both connectivity profiles are dissociated in different familiarity conditions, or whether they work in communion but assuming different roles in each context. A tentative interpretation is that both fronto-temporal and temporo-parietal theta connections are engaged during music listening, but positive valuation of the stimuli relies on fronto-temporal connectivity when music is novel and shifts its focus over temporo-parietal connectivity as music becomes familiar. Positive value would thus be assigned to efficient online predictive processing in the former case (schematic expectancy) and to efficient recognition in the latter (veridical expectancy). This latter case would be associated with a greater likelihood of reporting the most evoked pleasantness, considering our behavioral results and previous evidence^1^. It must be noted, however, that a variety of evidence exists showing how music-evoked pleasantness correlates with fronto-temporal activations using potentially familiar stimuli (e.g.^13,19^). Therefore, while we show a predominance of these connections during positive valuation of unfamiliar music, we cannot rule out its involvement in the valuation of familiar music.

It is also interesting to note that our results add up to several studies pointing out certain right hemispheric dominance in music processing (e.g.^19,24,41–44^), since all our results involve right temporal nodes, and frontal nodes are also right-lateralized. This right-hemispheric dominance could be attributed to the specialization of right temporal and frontal areas in pitch perception and auditory working memory, as well as in the detection of pattern violations^12^. However, evidence showing no hemispheric specialization also exists (e.g.^45^), as well as research noting that inter-hemispheric interactions are needed for normal music listening^46^. Therefore, we interpret our results as showing a relative right hemisphere dominance, rather than an absolute specialization.

The present study is not absent of limitations. While the use of EEG provided us with a good temporal resolution to study oscillatory dynamics, it lacks the appropriate spatial resolution to make more precise topological inferences, neither it captures the subcortical signals associated with the process of giving value to music. Multimodal experiments are necessary to replicate and link the results here present with reward signals in the striatum during peak pleasurable events, as well as to precise the cortical localization of the EEG signals. Other limitations include the fact that subjects evaluated the musical stimuli while listening to them, which might have imposed multitasking and an active listening strategy on participants^47^, and analyzing the data relative to the whole music fragments rather than studying the particular EEG, behavioral and acoustic time-courses, which may offer a complementary view on how the temporal dynamics of interest unfold (e.g. see^48–52^). Moreover, we only counted on one repetition and 24 h of consolidation to induce familiarity. While this resulted to be enough to observe familiarity effects and differences in the EEG data, more repetitions and tests would be necessary to see the extent to which these effects are stable or change over time. Finally, while we consider effective sample size (i.e. number of observations) sufficient for statistical inference in this study, generalizations of the results at the population level must be made cautiously. Larger sample sizes in similar studies will reveal the extent to which these results can be replicated.

## Conclusions

In the present experiment we studied how theta brain synchronization associated with music-evoked pleasantness is modulated by familiarity, showing how contextual factors influence musical preference and its underlying brain dynamics. Considering our results, the relation between music-evoked pleasantness and temporal theta synchronization is moderated by familiarity, with fronto-temporal connectivity being associated with pleasantness evoked by novel music and temporo-parietal connectivity with pleasantness evoked by familiar music, the latter being associated with greater reported pleasantness. These distinct mechanisms could be reflecting how positive valuation of musical stimuli shifts its focus from schematic expectations to veridical expectations as music becomes familiar. This claim must be furthered researched with the appropriate paradigms and methodology.

## Supplementary Information

Supplementary Information.
