# Supplementary material for: Different theta connectivity patterns underlie pleasantness evoked by familiar and unfamiliar music
Source: Sci Rep. 2021 Sep 17;11:18523. doi: 10.1038/s41598-021-98033-5 (PMC8448873; doi:10.1038/s41598-021-98033-5)
Supplement: Supplementary file 1 — Supplementary Information. [file 41598_2021_98033_MOESM1_ESM.docx]

**Different Theta Connectivity Patterns Underlie Pleasantness Evoked by Familiar and Unfamiliar Music: Supplementary Materials**

Alberto Ara^1,2^

Josep Marco-Pallarés^1,2^

*^1^Department of Cognition, Development and Educational Psychology, Institute of Neurosciences, University of Barcelona, Spain*

*^2^Cognition and Brain Plasticity Unit, Bellvitge Biomedical Research Institute, L'Hospitalet de Llobregat, Spain*

| **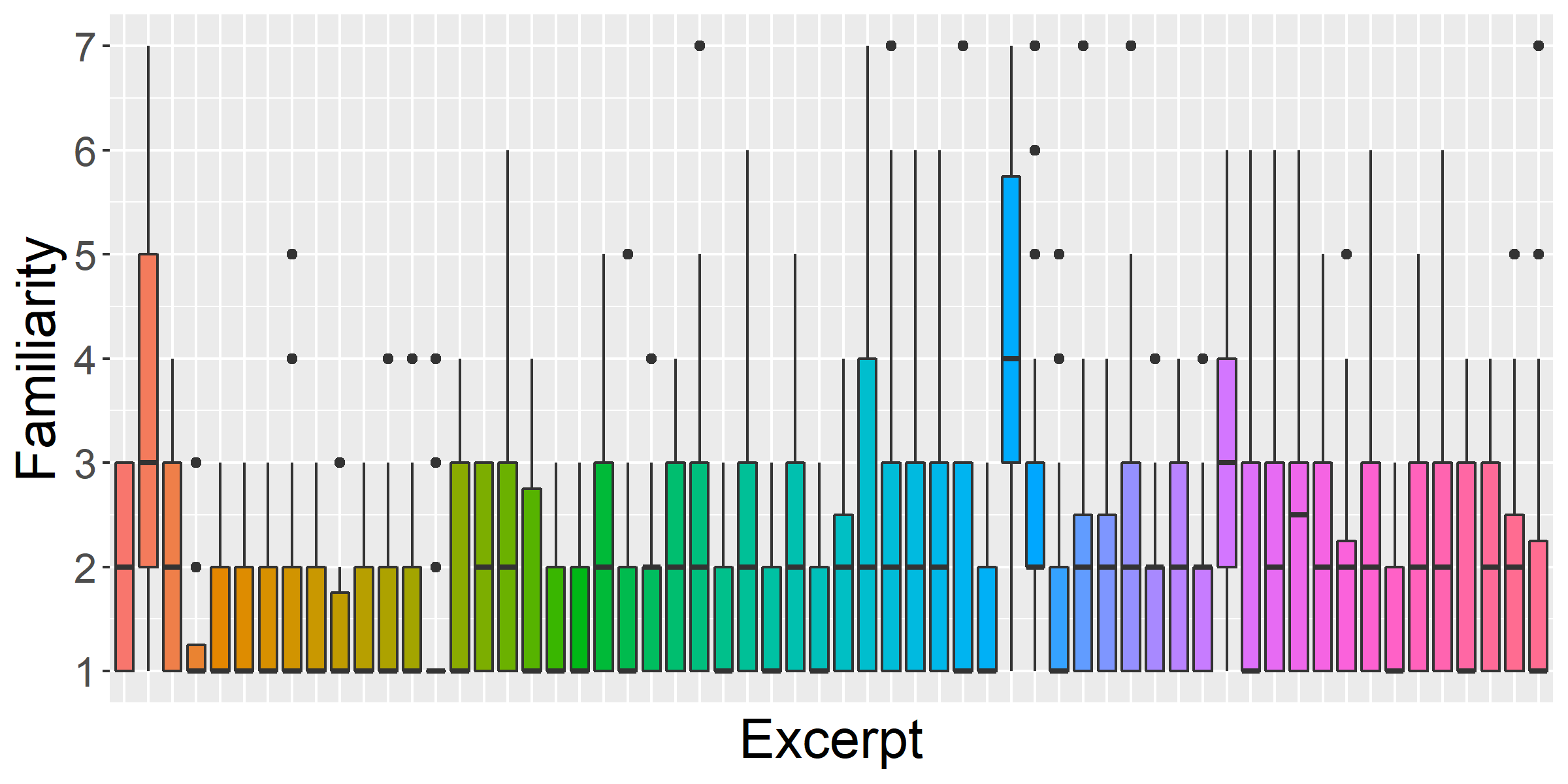** |
| --- |
| **Fig S1.** Distribution of familiarity ratings per excerpt the first time they were listened to (exposure session). Plot generated in R [53] with package ggplot2 [54]. |
| **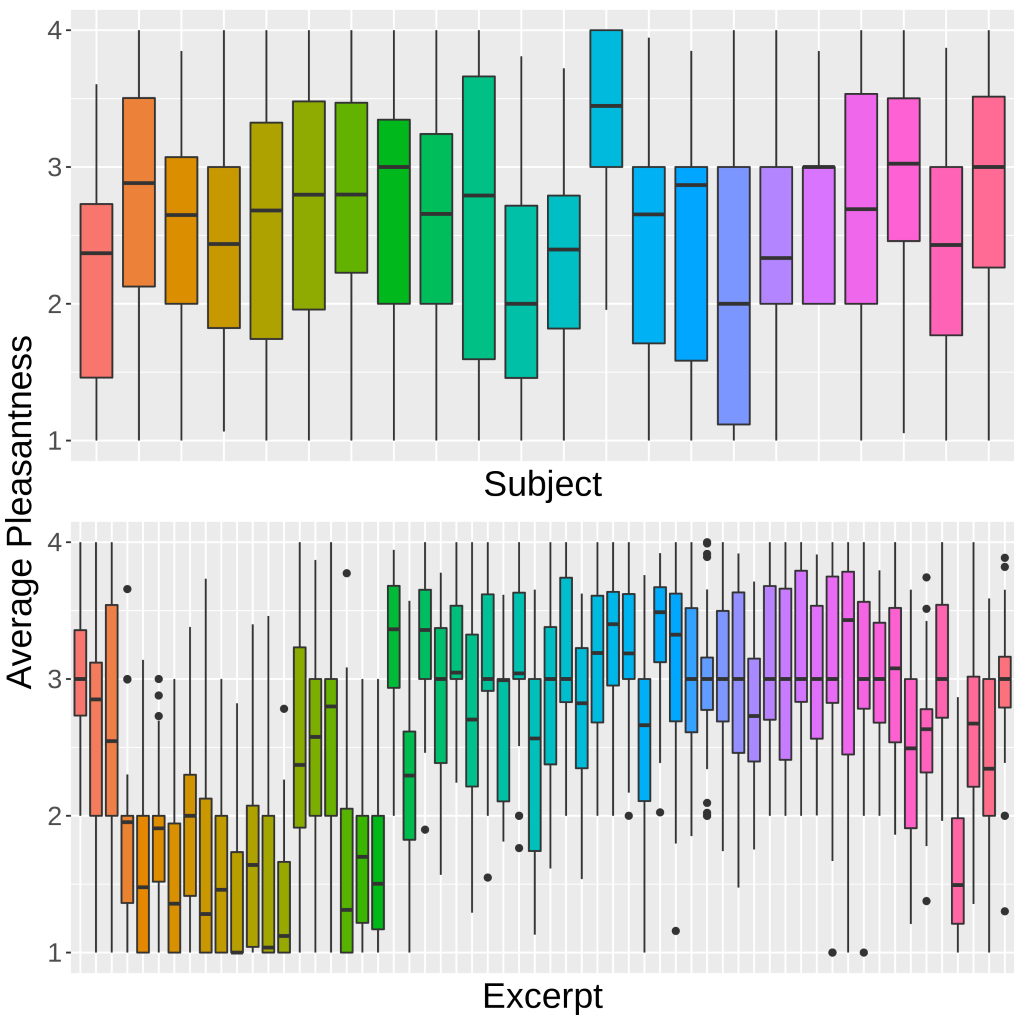** |
| **Fig S2.** Distribution of time-weighted self-reported pleasantness per subject (top) and excerpt (bottom). Plot generated in R [53] with package ggplot2 [54]. |

|  | ***A) most liked ≠ least liked***  ***(new music)*** | ***B) most liked ≠ least liked***  ***(old music)*** |
| --- | --- | --- |
| p < 0.05 | 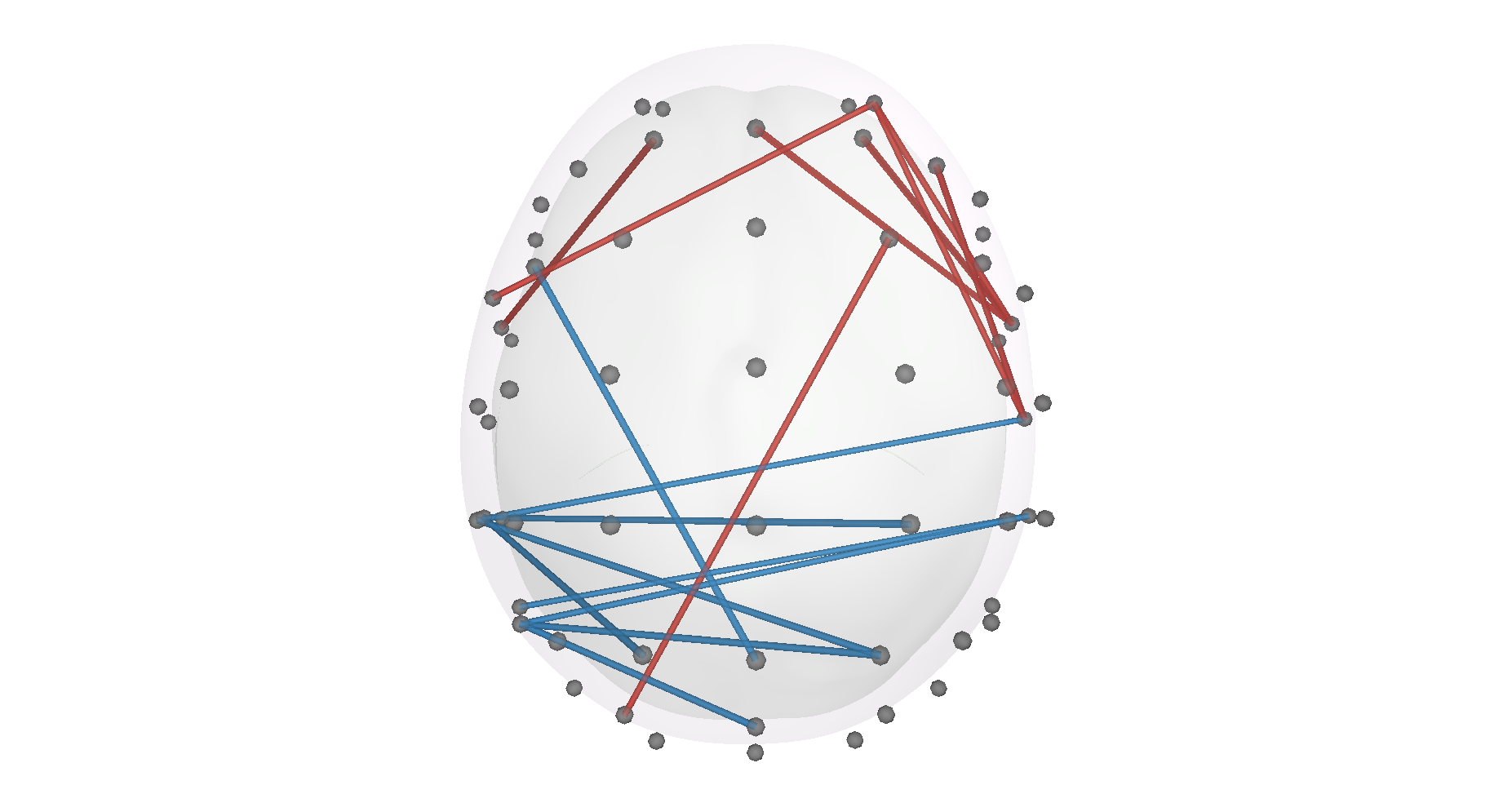 | 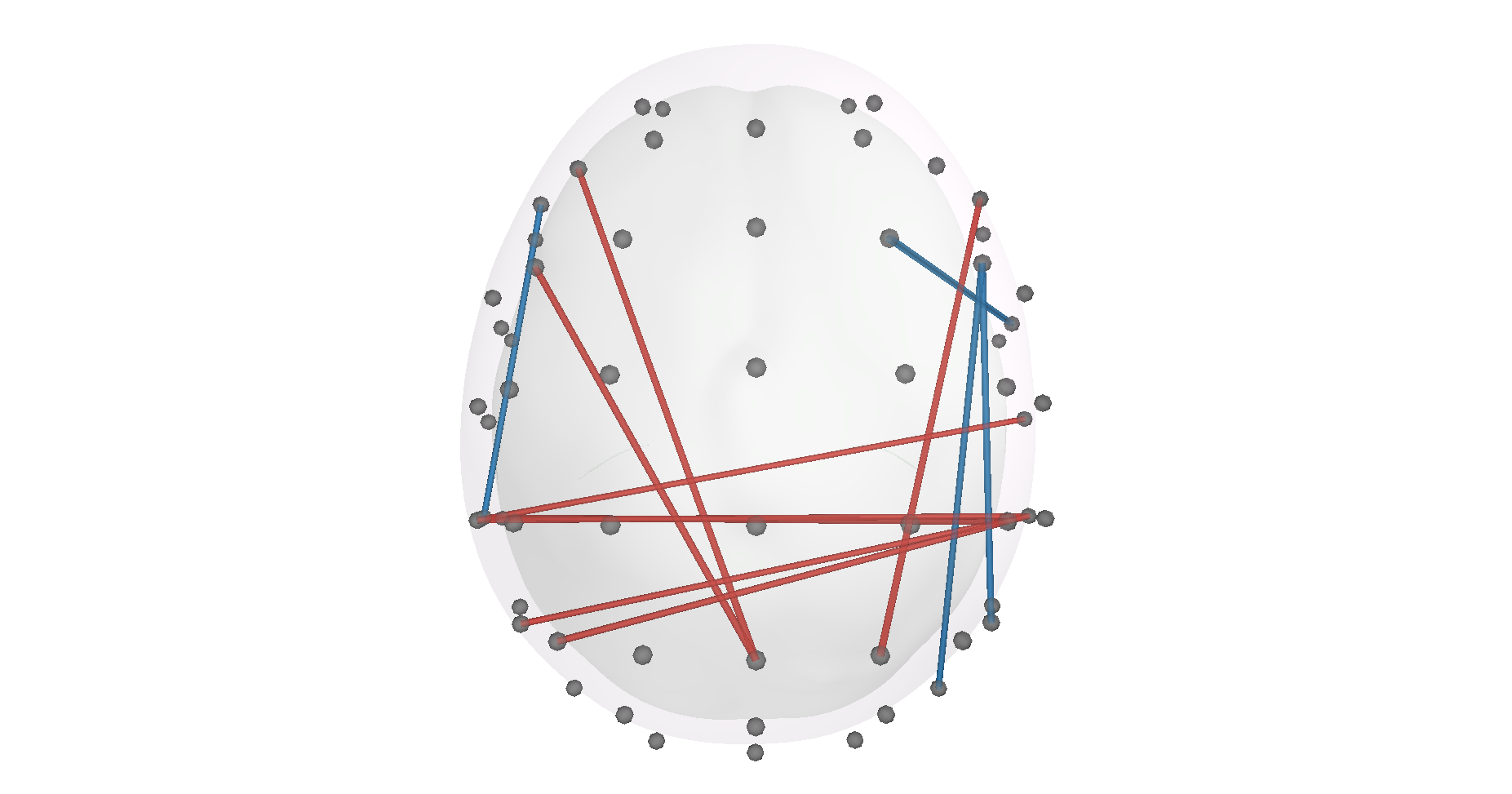 |
| p < 0.01 | 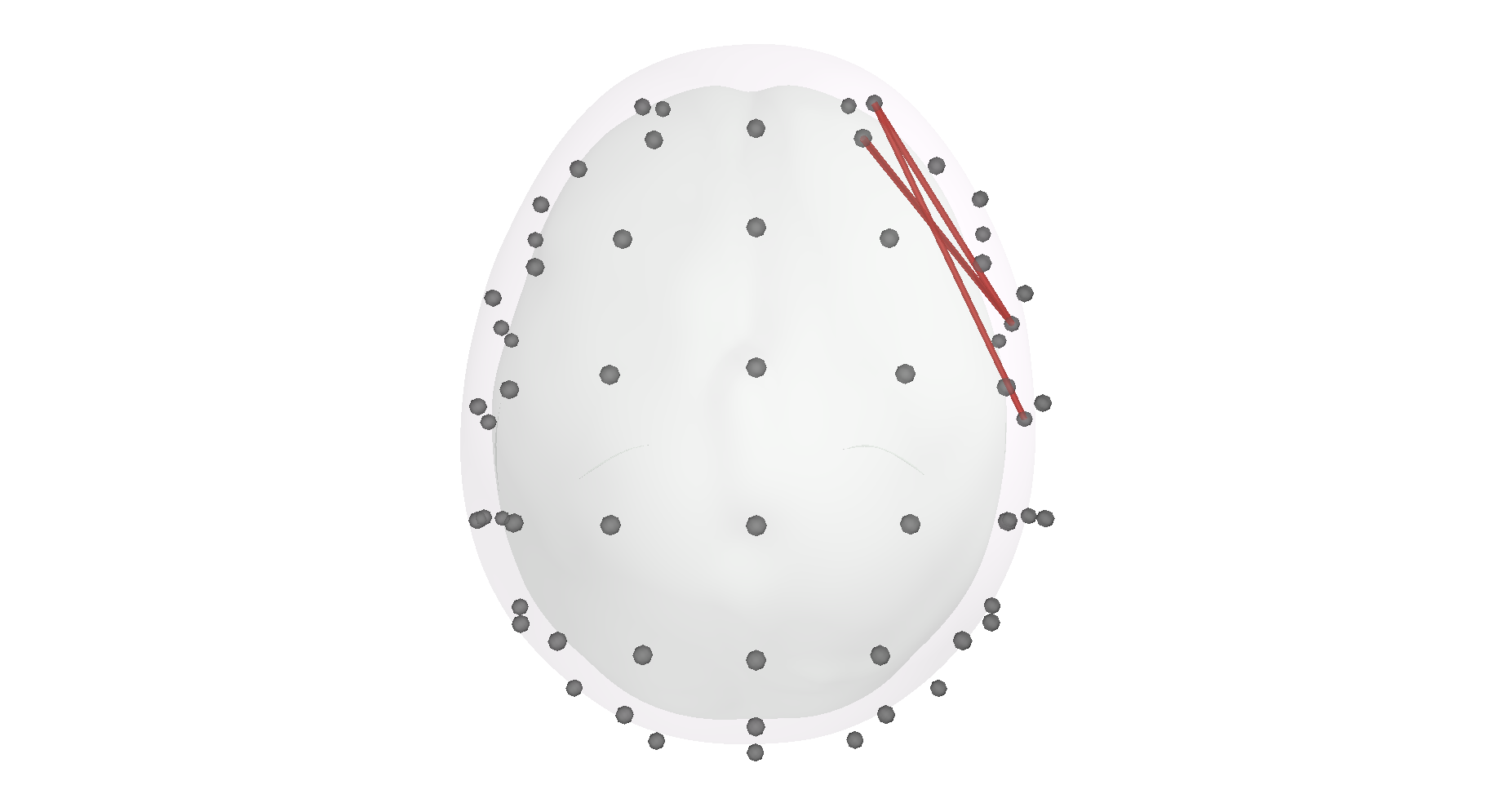 | 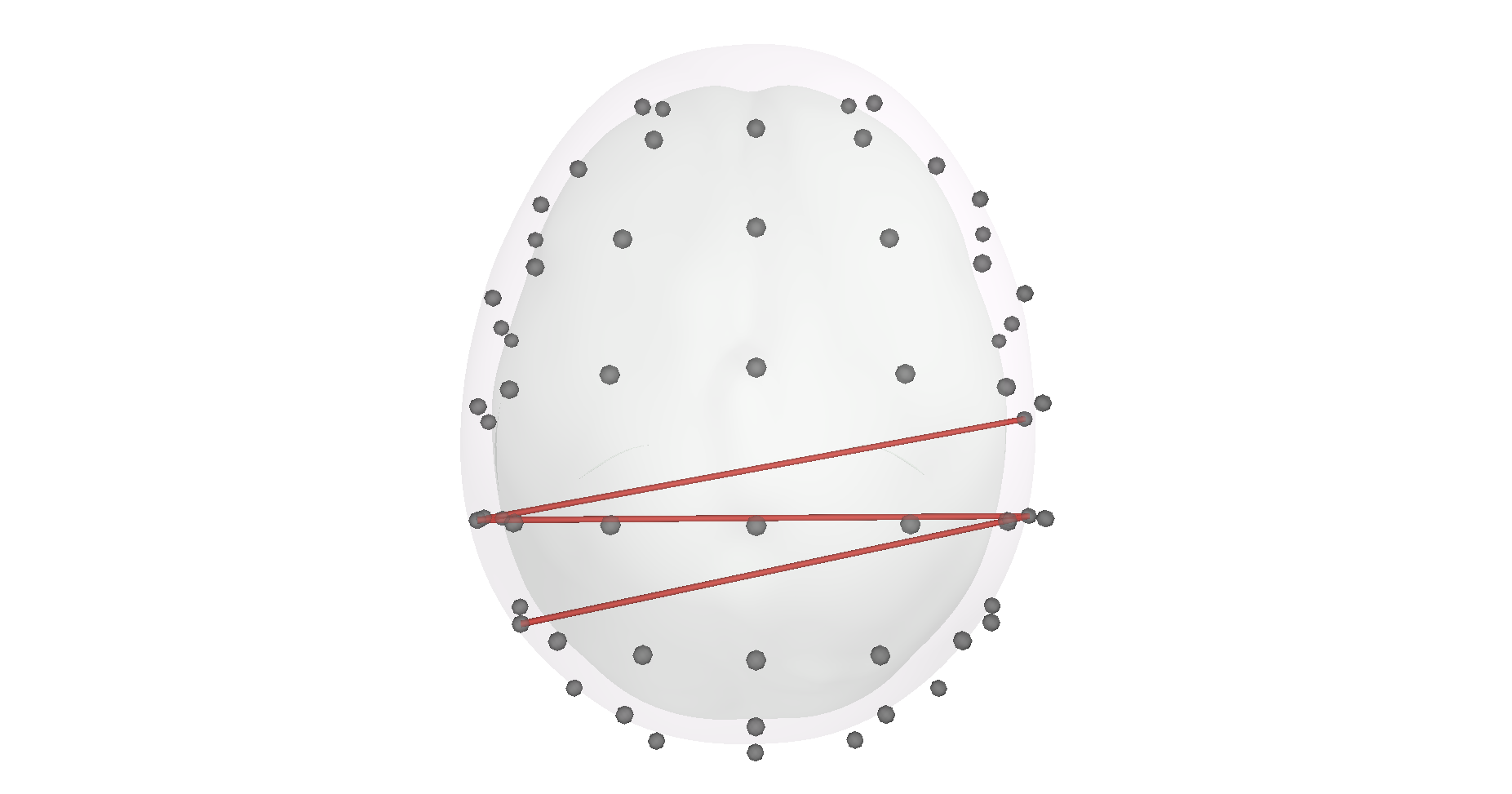 |
| p < 0.005 | 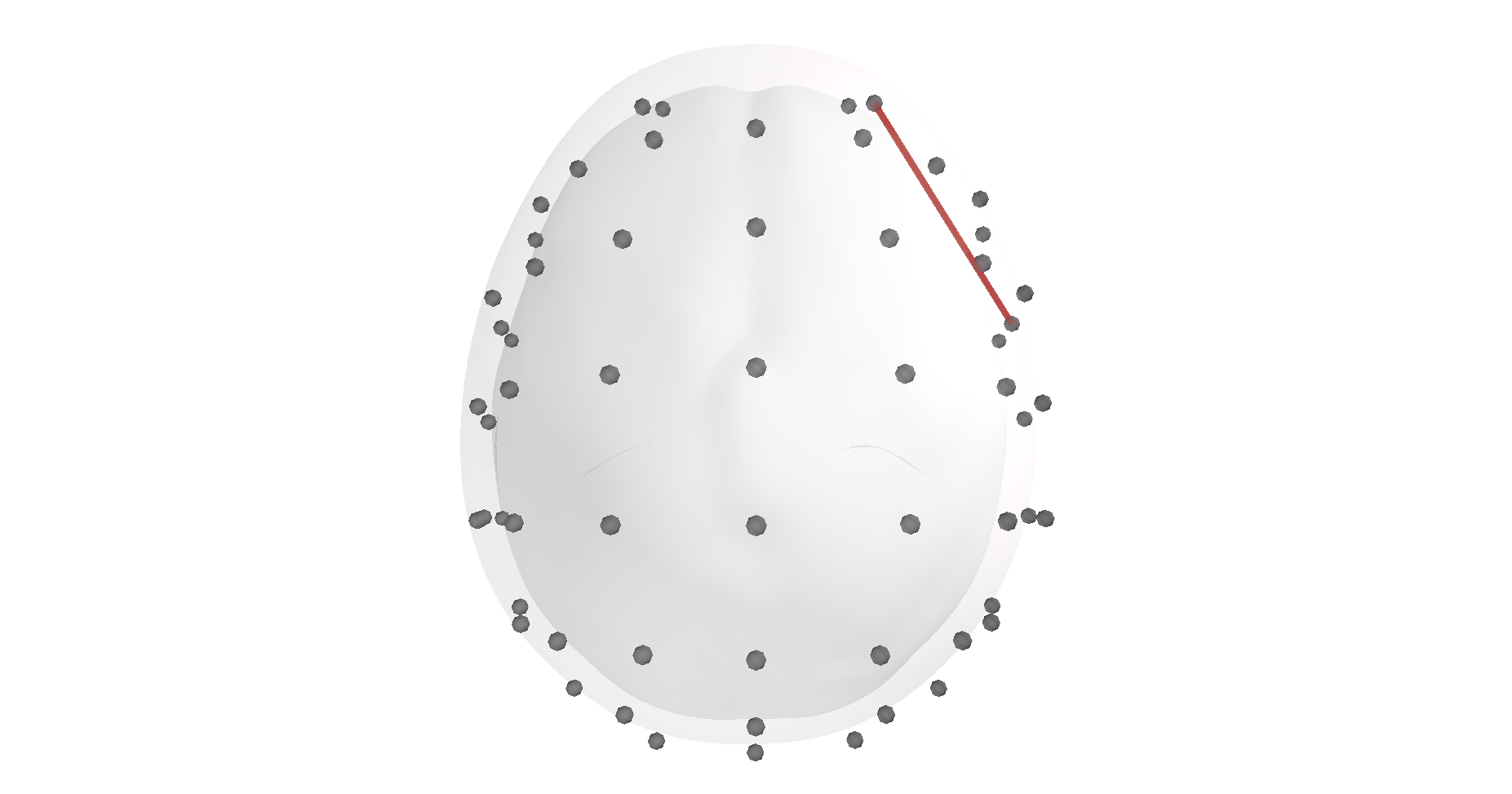 | 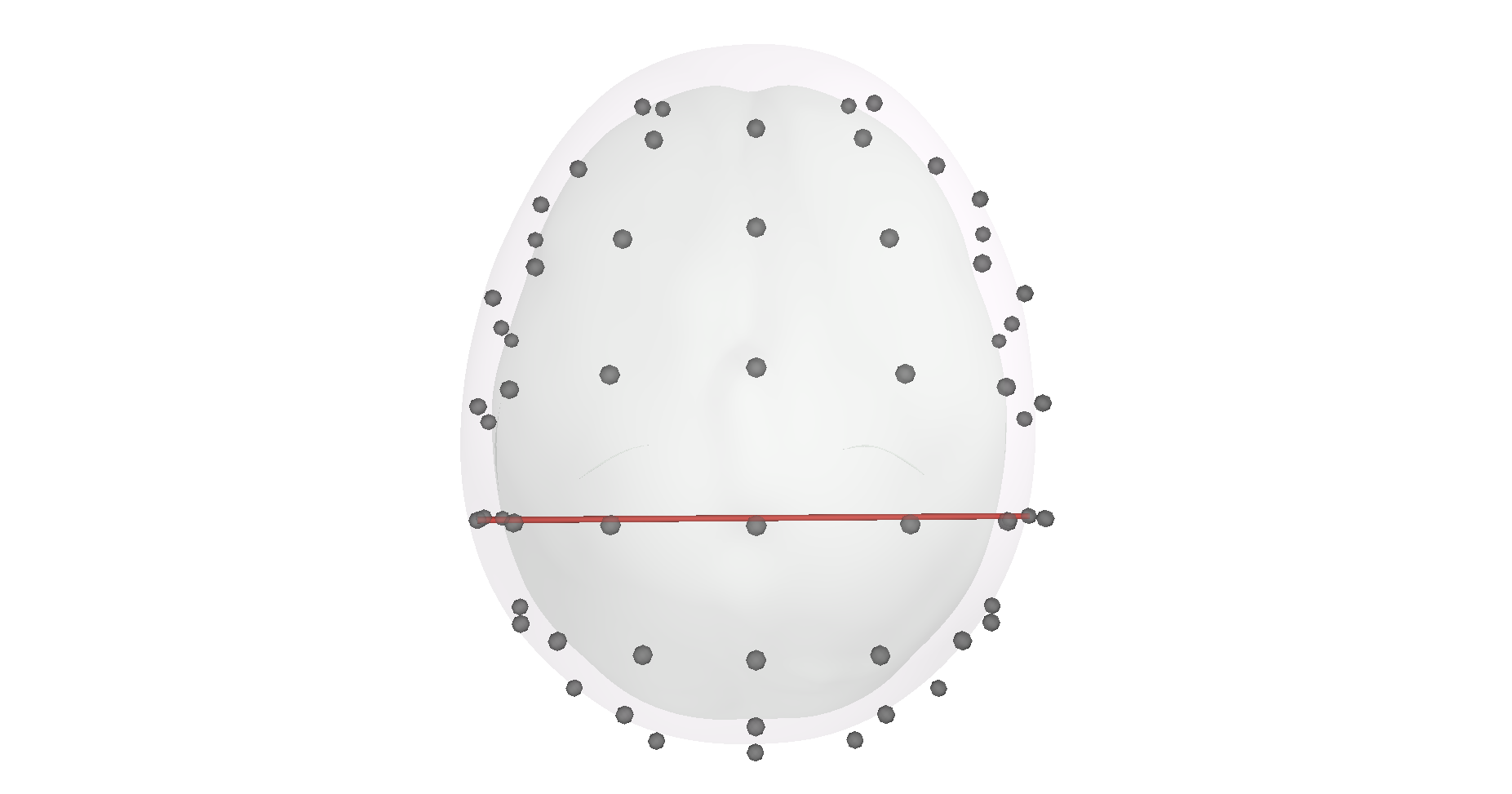 |
| p < 0.001 |  | 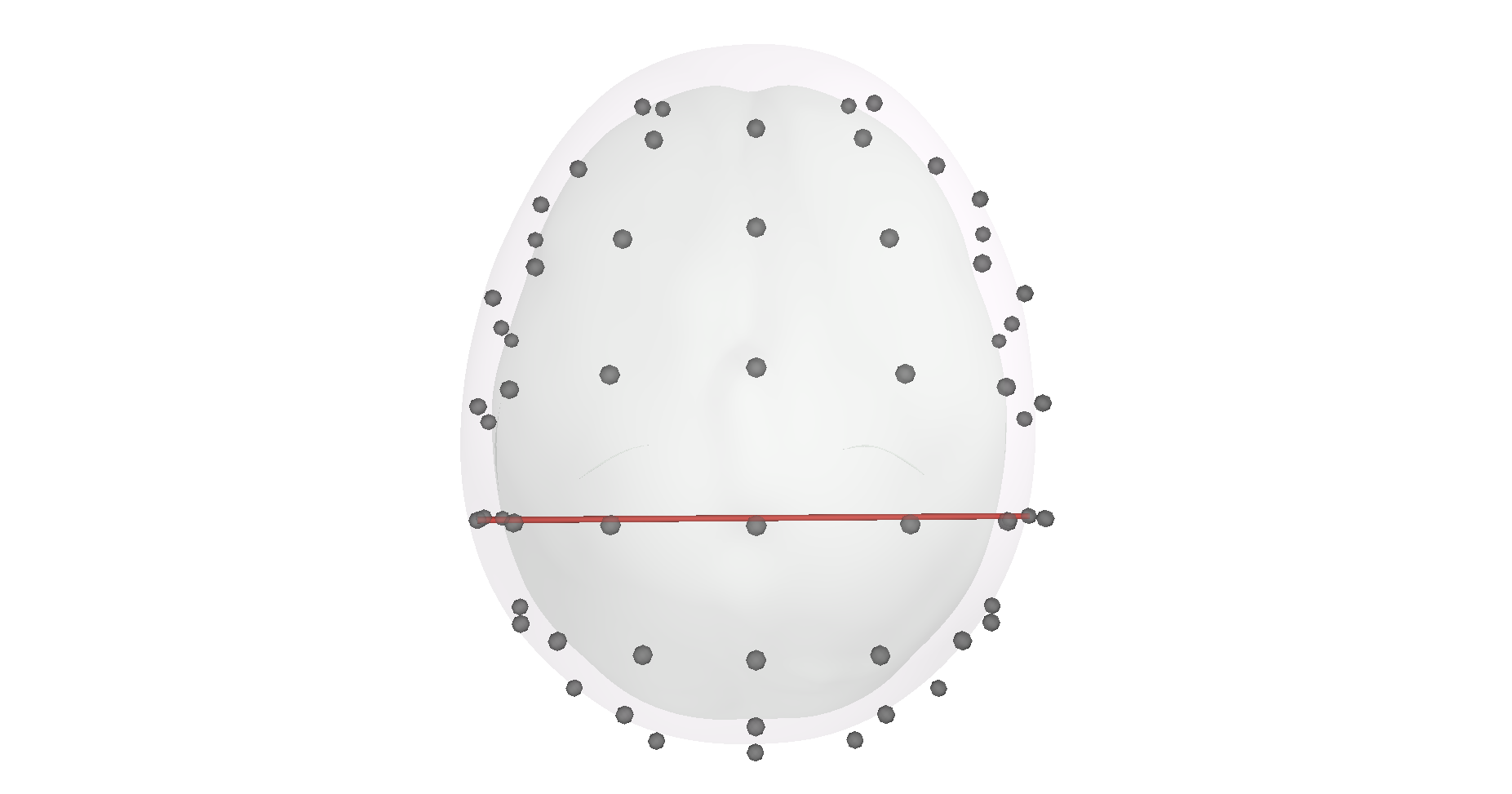 |
| **Fig S3.** Significant connections in theta under the frequentist framework with different alpha levels for **A)** effects of liking for novel music and **B)** effects liking for familiar music. Red lines indicate positive relationships and blue lines negative relationships. No connection survived family-wise error or false discovery rate corrections. Models were implemented with R package glmmTMB. | | |

| **Artist** | **Track** | **Passage(s)** |
| --- | --- | --- |
| Anjulie | Boom | Verse, Chorus |
| Atari Teenagers | New Blood | Intro, Verse |
| Atari Teenagers | Street Grime | Intro, Verse |
| Basshunter | All I ever wanted | Pre-chorus, Chorus |
| Baz Corden | Hearts and Rainbows | Verse, Chorus |
| Blinding | The Island | Beat |
| Dub Trees | Buffalo | Beat, Beat |
| Caro Emerald | Just One Dance | Verse, Chorus |
| Cashmere Cat | Mirror Maru | Beat, Break, Beat |
| Comforce | Semantic Field | Beat |
| Conrad Schnitzer | Wurm | Beat |
| Daft Punk | The Game Has Changed | Beat |
| Dua Lipa | Blow Your Mind (Instrumental) | Pre-chorus, Chorus |
| Errors | A Rumor in Africa | Intro, Verse |
| Finally Awake | Gone Away | Verse, Chorus |
| Florence + The machine | Dog days are over (instrumental) | Verse, Chorus |
| James Arthur | Coming Home for Summer | Verse, Chorus |
| James Welsh | Nowt | Intro, Beat |
| JRY ft. Rooty | Pray | Pre-chorus, Chorus |
| Kaskade ft. Martina of Dragonette | Fire your new shoes | Verse, Chorus |
| Klingande | Jubel (original mix) | Beat |
| KSHRM | Kashmir (original mix) | Break, Beat |
| Linkin Park | Heavy (instrumental) | Verse, Chorus |
| Linkin Park | Battle Symphony (instrumental) | Pre-chorus, Chorus |
| Marina & the Diamonds | Hollywood (instrumental) | Verse, Pre-chorus, Chorus |
| Martin Solveig & GTA | Intoxicated (instrumental) | Intro, Beat |
| Martin Solveig ft. Sam White | +1 | Verse, Beat, Verse |
| Maybeshewill | Opening | Solo |
| Metric | Combat Baby (instrumental) | Verse, Chorus |
| Metric | Speed the collapse | Verse, Chorus |
| **Table S1.** List of songs used in the experiment and the corresponding passages used in the excerpts. Songs labeled as instrumental are instrumental versions of songs originally written with lyrics. | | |

| **Artist** | **Track** | **Passage(s)** |
| --- | --- | --- |
| Moguai ft. Cheat Codes | Hold On | Verse, Beat |
| Mumdance | Shook | Verse |
| Mumdance | Take time | Verse |
| Nine Inch Nails | 8 ghosts I | Beat |
| Nine Inch Nails 1 | 14 ghosts II | Beat |
| Núria Graham | Unknown | Verse, Chorus |
| Paloma Faith | Black & Blue | Verse, Chorus |
| Paloma Faith | Only Love Can Hurt Like This (instrumental) | Verse |
| Passenger | Bullets | Chorus |
| Quarta 330 | Digital lotus flower | Intro, Beat |
| Richard Devine | Pallete | Beat |
| Richard Devine | Reneanalogueseq | Beat |
| Robin Bengtsson | Constellation Prize | Pre-chorus, Chorus |
| Robin Dtjiernberg | Rain (Didrick remix) | Pre-chorus, Beat |
| Roco | Dealer | Verse, Chorus |
| S-Type | Billboard (Lido Remix) | Break, Beat, Break |
| Scooter | Maria (I like it loud) | Beat |
| Scooter | My gabber | Break, Beat, Break |
| Sia | Alive (instrumental) | Verse, Pre-Chorus, Chorus |
| Special D | Come with me | Pre-chorus, Chorus |
| Special D | You | Verse, Break, Beat |
| Stargate ft. Pink & Sia | Waterfall (instrumental) | Verse, Chorus |
| Stephen Marley | Hey Baby (instrumental) | Verse |
| Subshock Evangelos | All Right | Break, Beat |
| The Mad Trist | Pay the Piper | Verse, Chorus |
| TNGHT | Goooo | Intro, Beat, Break, Beat |
| Triana Park | Iron Blue | Pre-chorus, Chorus |
| Ulver | Darling, didn’t we kill you? | Beat, Outro |
| Venetian Snares | Öngyilkos Vasárnap | Beat |
| ZAYN | Fool For You (instrumental) | Verse, Break, Chorus |
| **Table S1 (continuation)** | | |
